# The evolution of TAVI performance overtime: an overview of systematic reviews

**DOI:** 10.1186/s12872-024-03980-2

**Published:** 2024-06-21

**Authors:** Carmen Angioletti, Giaele Moretti, Stefania Manetti, Luigi Pastormerlo, Milena Vainieri, Claudio Passino

**Affiliations:** 1 Management and Healthcare Laboratory, Institute of Management, Sant’Anna School of Advanced Studies, Pisa, Italy; 2 Department of Management Engineering, Politecnico di Milano, Milano, Italy; 3 Fondazione Toscana G. Monasterio, Pisa, Italy; 4 Health Science Interdisciplinary Center, Sant’Anna School of Advanced Studies, Pisa, Italy

**Keywords:** Transcatheter aortic valve replacement, Surgical aortic valve replacement, Review

## Abstract

**Background:**

Transcatheter aortic valve implantation (TAVI) is a well-established treatment for high and intermediate-risk patients with severe aortic stenosis (AS). Recent studies have demonstrated non-inferiority of TAVI compared to surgery in low-risk patients. In the past decade, numerous literature reviews (SLRs) have assessed the use of TAVI in different risk groups. This is the first attempt to provide an overview of SRs (OoSRs) focusing on secondary studies reporting clinical outcomes/process indicators. This research aims to summarize the findings of extant literature on the performance of TAVI over time.

**Methods:**

A literature search took place from inception to April 2024. We searched MEDLINE and the Cochrane Library for SLRs. SLRs reporting at least one review of clinical indicators were included. Subsequently, a two-step inclusion process was conducted: [1] screening based on title and abstracts and [2] screening based on full-text papers. Relevant data were extracted and the quality of the reviews was assessed.

**Results:**

We included 33 SLRs with different risks assessed via the Society of Thoracic Surgeons (STS) score. Mortality rates were comparable between TAVI and Surgical Aortic Valve Replacement (SAVR) groups. TAVI is associated with lower rates of major bleeding, acute kidney injury (AKI) incidence, and new-onset atrial fibrillation. Vascular complications, pacemaker implantation, and residual aortic regurgitation were more frequent in TAVI patients.

**Conclusion:**

This study summarizes TAVI performance findings over a decade, revealing a shift to include both high and low-risk patients since 2020. Overall, TAVI continues to evolve, emphasizing improved outcomes, broader indications, and addressing challenges.

**Supplementary Information:**

The online version contains supplementary material available at 10.1186/s12872-024-03980-2.

## Background

Aortic stenosis (AS) is a condition caused by the narrowing of the aortic valve and is one of the most common valvular diseases. The prevalence of severe aortic stenosis in subjects aged ≥ 75 years is about 3.4% in Western countries [1]. Resulting symptoms include shortness of breath, chest pain, fatigue, and reduced exercise tolerance. If left untreated, AS can lead to heart failure and death [2]. For decades, Surgical Aortic Valve Replacement (SAVR), which involves an open-heart surgery in which the diseased aortic valve is replaced with a prosthetic valve, has been considered the gold standard treatment [3]Transcatheter aortic valve implantation (TAVI) is a minimally invasive procedure that has progressively emerged as a valid treatment option in prohibitive, high, and intermediate-risk patients [4] respectively. More recently, randomized trials have demonstrated non-inferiority of TAVI compared to surgery in low-risk patients as assessed by the Society of Thoracic Surgeons (STS) score. TAVI is performed by percutaneous delivery of a bioprosthetic valve through a catheter typically inserted via the femoral artery. Unlike SAVR, TAVI can be performed under local anaesthesia, and conscious sedation with minimalist vascular access. Over the past decade, an increasing number of systematic reviews (SRs) have been conducted to validate the extent of the indication for TAVI in low-risk patients. This is the first effort to summarize the evidence in an overview of SRs (OoSRs) focusing exclusively on secondary studies that have reported at least one review of clinical outcomes/process indicators. The aim of this research is to summarize the findings of extant literature reviews on the performance of TAVI over time. This study provides a useful overview of the main Key Performance Indicators (KPI) such as early and late mortality, discussing the evolution of TAVI in terms of the target population and the related results over time to highlight whether and where there are uncertain findings requiring further investigation.

## Methods

### Data searches and study selection

The authors declare that all supporting data are available within the article. This work constitutes an umbrella approach to identifying both SLRs and RCTs.

A purposive literature search for SLRs took place from the beginning (2013) to January 2023. We searched MEDLINE (via PubMed) and the Cochrane Library for SRs. A more comprehensive search strategy was applied in MEDLINE, using the medical subject heading (Appendix I in the Data Supplement). For all included studies, reference lists were also searched.

Four independent overview authors (SM, LP, GM, and CA) screened the titles and abstracts against the eligibility criteria for inclusion.

Search strategy: (“TAVI“[Title/Abstract] OR “transcatheter aortic valve implantation“[Title/Abstract] OR “TAVR“[Title/Abstract] OR " transcatheter aortic valve replacement “[Title/Abstract] OR “transcatheter aortic valve replacement“[MeSH Terms]) AND (“Review“[Title/Abstract] OR “meta-analysis“[Title/Abstract] OR “meta-analysis as topic“[MeSH Terms])

We searched for SLRs that compared the efficacy and safety of TAVI in patients with aortic stenosis and analyzed the full texts to extract the eligible RCTs. SLRs that included observational studies were excluded. Studies that did not use SAVR in the control group were excluded. Narrative reviews that did not report any search strategy or that did not critically appraise the included studies’ quality were excluded, as well as studies that focused only on devices or imaging. Only studies considering the first intervention were included, with VIV-TAVI and redo-SAVR interventions excluded.

Finally, another reviewer intervened when there was a disagreement between the authors.

### Inclusion process

SLR reporting at least one review of clinical effectiveness/complication indicators have been considered for inclusion.

### Step 1 – screening of titles and abstracts

Titles and abstracts of the references found (*n* = 2,483) were screened independently by meta-reviewers (CA, SM, GM, and LP), to check whether these publications satisfied the inclusion criteria. In this phase, the two reviewers agreed in virtually 100% of the cases. For the references selected by both reviewers and those selected by only one reviewer (a total of 85), we tried to track down or download the full text.

### Step 2 – screening based on full texts

Next, the full texts were assessed independently by the first and second meta-reviewer (CA and SM or GM) using the inclusion criteria cited. In this phase, we conducted a manual search in the reference lists of the full-text papers.

The full texts of these additional references were studied as well, which brings the total number of full texts examined to 96.

### Data extraction and Quality Assessment

Four reviewers (SM, LP, GM, and CA) independently extracted data/items including citation details; objectives of the included review; type of review; participant details; setting and context; the number of databases sourced and searched; date range of database searching; the number of studies, types of studies and country of origin of studies included in each review; instrument used to appraise the primary studies and the rating of their quality; outcomes reported that are relevant to the overview of systematic literature reviews (OoSRs) question; method of synthesis/ analysis used to synthesize the evidence; major conclusions; comments or notes by the OoSRs authors; metric used and effect size (for meta-analyses); Coefficient intervals Cis (for meta-analyses). Authors were contacted in case of missing data. If the requested data could not be retrieved, the study was not included in the analysis. A Measurement Tool to Assess Systematic Reviews (AMSTAR) [5] was adopted to perform a rapid and reproducible critical assessment of the methodological quality of the included studies. Finally, studies whose methodological quality was judged to be critically low according to the AMSTAR assessment tool were excluded.

## Results

**Fig. 1:**
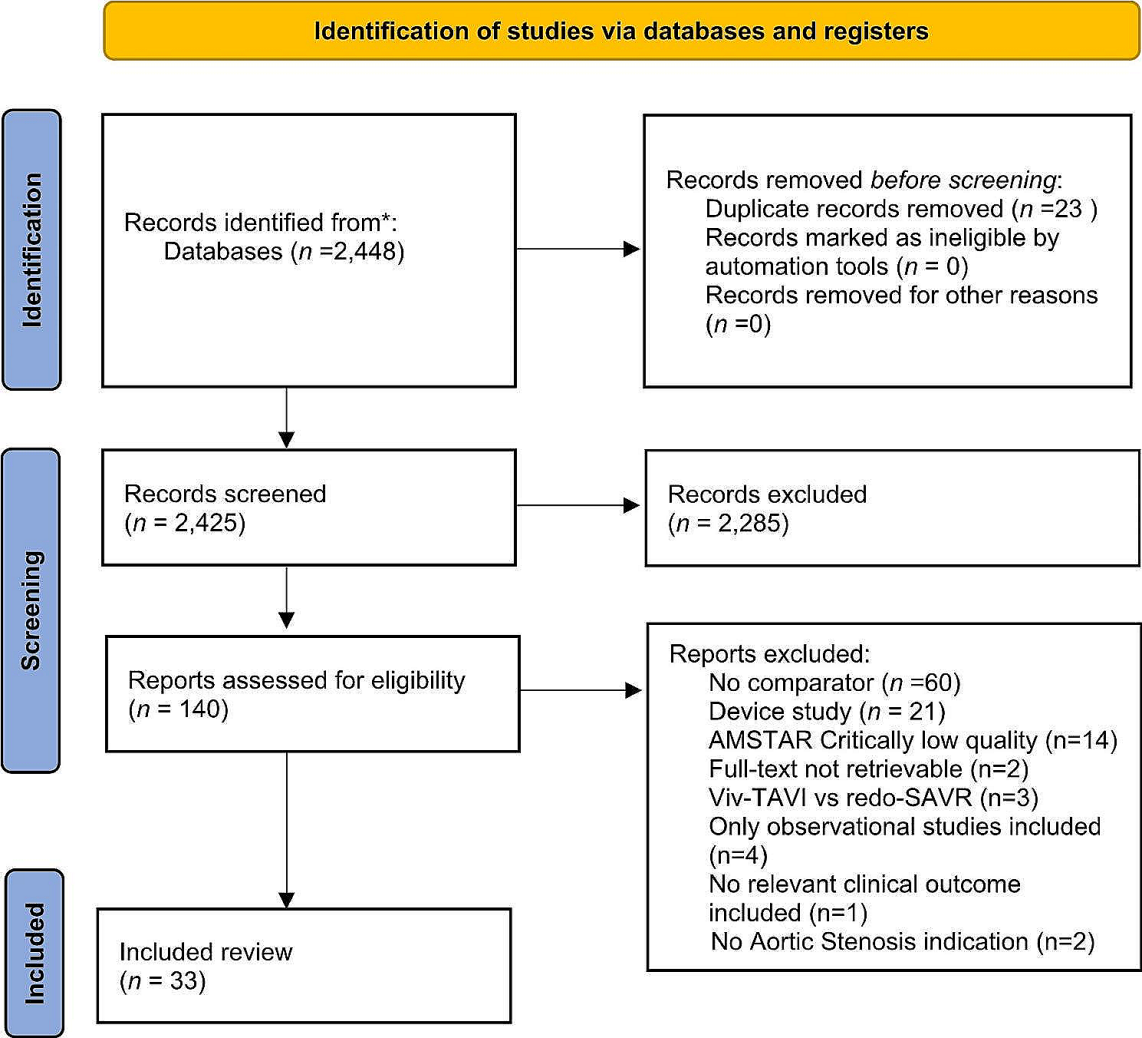
Workflow for the selection of eligible studies following the Preferred Reporting Items for Systematic Reviews and Meta-Analyses (PRISMA) criteria

Figure 1 summarizes the screening process. We found 33 SLRs (Appendices 2 in the Data Supplement). All the included studies were published between 2013 and April 2024 in English. Overall, 22 SLRs collected their data only from Randomized Controlled Trials (RCTs), whereas 11 SLRs collected data from randomized and non-randomized studies and prospective or retrospective observational studies.

**Fig. 2:**
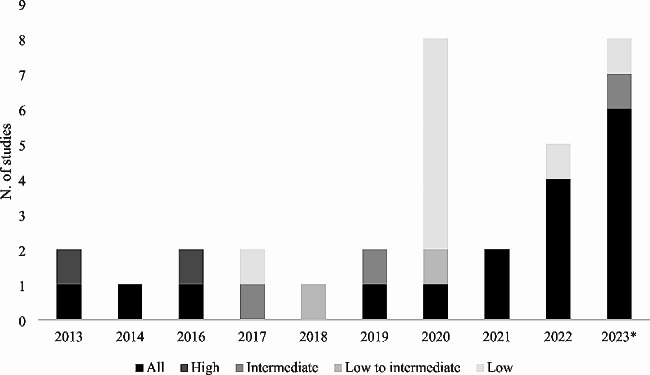
Distribution of risk classes included in studies per different years. The figure reports the number of studies including All risks of patients, high, intermediate, low to intermediate, and low risk distribution per year. *The year 2023 includes also one paper published in 2024

Twenty-three studies considered elderly patients, over 65 years old. Sixteen studies included all surgical STS-risk patients. Low, low to intermediate, intermediate, and high risk were considered in 9, 2, 3, and 2 studies respectively. Figure 2 shows the evolution of the risk class of patients included in studies over time. Table 1 summarizes clinical outcomes results for TAVI compared to SAVR, whereas Table 2 summarizes complications regarding included studies.

### Clinical outcomes

#### Mortality

##### Early mortality

Early mortality is defined as mortality at 30 days or in-hospital in fourteen studies [6–19], as 3-month mortality in one study [20] and as 6-month mortality in another study [21]. Only one study did not define the time interval considered [22].

Early mortality was evaluated in eighteen out of the thirty-three studies included. Ten studies [6, 8, 9, 13, 15–18, 20, 22] found early mortality to be similar in both TAVI and SAVR groups. Five studies [10, 11, 14, 19, 21] found TAVI to be associated with lower rates of early mortality. In one study [7], early mortality for TAVI vs. SAVR was found to be lower in patients with chronic kidney disease (CDK), whereas another study [12] assessed better outcomes for TAVI vs. SAVR in women. Lastly, one study reported lower rates of early mortality for low-risk patients and similar rates for high-risk patients [23].

##### Late Mortality

Late mortality was identified with mortality at 1 year of follow-up for most of the studies included [6, 8, 10–12, 15, 16, 20, 23–25] whereas five studies [13, 17, 18, 21, 26] considered two years. One study [19] reported late mortality for a follow-up period of 5 years. Two studies [14, 17] did not report the time interval for this outcome, and one study [22] considered more than 1 year.

Late mortality was evaluated in twenty out of the thirty-three studies included and was found to be similar between TAVI and SAVR in eleven studies [6, 8, 9, 11, 15, 17, 18, 20, 22–26]. One study [12] reported lower mortality rates for TAVI in medium-risk women. Two studies [10, 16] found TAVI to be associated with lower mortality overall, considering low surgical-risk patients. Four studies [13, 14, 19, 21] favoured SAVR over TAVI, all three including patients with all surgical risks. One study [23] found mortality to be lower for TAVI in patients with high surgical risk and similar to SAVR for low surgical risk patients.

##### All-Cause mortality

Eleven studies evaluated all-cause mortality; four [24, 27–29] found that it was similar between TAVI and SAVR, whereas two studies [30, 31] reported a lower rate for TAVI in the first year and similar rates in the second year of follow-up. Two studies favored SAVR [32, 33], and one study [34] reported a lower mortality for TAVI in the first follow-up year, which becomes equal to SAVR in the second year until it worsens between years two and five.

##### Cardiac mortality

Cardiac mortality was specifically evaluated in six out of the thirty-three studies included. Four [18, 24, 27, 35] studies found cardiac mortality to be similar between TAVI and SAVR. Two studies [29, 36] favored SAVR over TAVI and one [30] reported a lower rate for TAVI in the first year and similar rates in the second year of follow-up. One study [34] reported similar rates in the first year of follow-up, higher rates in the second year for TAVI, and similar rates until year five of follow-up.

#### Stroke and transient ischemic attacks (TIA)

Twenty-four studies out of the thirty-three selected evaluated the risk of stroke. Sixteen studies [6, 8, 11, 13–16, 22, 24, 26–29, 34, 36–38] found the hazard for stroke to be similar between TAVI and SAVR. Two studies [9, 18] found TAVI to have a higher rate of stroke than SAVR, with the first study considering only high-risk surgical patients and the second one including patients with all risk classes. Four studies [21, 25, 31, 39] found TAVI to be associated with a lower incidence of stroke. Of those, three included patients with all classes of surgical risk and one included only low surgical risk patients.

One study [7] found TAVI to be favorable in patients with CDK, whereas another one [23] reported lower incidence in patients with low surgical risk and similarities between TAVI and SAVR in high surgical risk patients.

Two studies out of the thirty-three selected evaluated the risk of developing TIA, one [18] found similarities between TAVI and SAVR, whereas the other one reported a lower rate of TIA with TAVI.

#### Myocardial infarction

Thirteen studies out of the thirty-three selected evaluated the occurrence of periprocedural myocardial infarction. Ten studies [6, 8, 11, 13–16, 18, 26, 29] reported similarities between TAVI and SAVR. Three studies [20, 27, 31] found TAVI to be associated with lower rates of myocardial infarction.

### Complications

#### Major bleeding

Sixteen studies out of the thirty-three selected evaluated the risk of significant bleeding complications. Twelve studies [8–10, 16, 18, 22, 25, 26, 28–30, 35, 37] found TAVI to be associated with lower rates of major bleeding. One study [23] found TAVI to be less favourable than SAVR and two studies [6, 13] reported similarities between TAVI and SAVR.

One review [8] reported that, in the early days of TAVI, bleeding was the most common complication resulting from the use of large bore sheaths. However, advancements in percutaneous techniques, ultrasound guided vascular cannulation, smaller catheter systems, and operator experience have reduced periprocedural bleeding in TAVR over time.

#### Acute kidney injury (AKI)

Eighteen studies out of the thirty-three selected evaluated the risk of developing acute kidney injury. Sixteen studies [7, 8, 10, 11, 13, 14, 16, 20, 22, 25–30, 35] found TAVI to be associated with a lower incidence of AKI. One study [15] found similarities between TAVI and SAVR. One study [23] reported a higher incidence of AKI in TAVI patients.

#### Vascular complication

Twelve studies out of the thirty-three selected evaluated the risk of developing vascular complications. Nine studies [9, 11, 13, 14, 16, 20, 22, 25, 29] found TAVI to be associated with a higher incidence of vascular complications. One study [27] reported similar results between TAVI and SAVR, and another study [7] was neutral in patients with CDK. One study [23] favoured TAVI over SAVR for the development of vascular complications.

#### Pacemaker implantation

Twenty-two evaluated the rate of pacemaker implantation after the procedure. Eighteen studies [6–17, 22, 25–30] found TAVI to be associated with a higher risk of pacemaker implantation (PI), whereas one [24] found similarities between TAVI and SAVR. One study [23] reported TAVI to be associated with lower rates of pacemaker implantation. One study found TAVI to be associated with a lower risk of PI in the first two years after the operation, and similar rates from year 20 to year 5.

Subgroup analyses were performed by some authors to discern the risk of pacemaker implantation by valve type, comparing Self-Expanding VALVES (SEV) versus Balloon-Expandable (BEV) valves.

Four studies [6, 10, 11, 27] found that within TAVI procedures, BEV had a lower incidence of PI, whereas one study [17] found that mechanically-expandable valve (MEV) was associated with an increased risk of pacemaker implantation compared to BEV, SEV, and SAVR in the long term.

#### Residual aortic regurgitation and moderate-severe paravalvular leak

Seven studies [6, 9, 15, 16, 22, 25, 27] out of the thirty-three selected evaluated the complication of residual aortic regurgitation, all reported higher rates associated with TAVI interventions.

Six studies out of the twenty-seven selected evaluated moderate-severe paravalvular leaks. All six [12, 15, 16, 20, 24, 28] reported TAVI to have a higher incidence than SAVR, with one study [12] detailing a better outcome specifically for male patients.

#### Atrial fibrillation

Eleven studies out of the thirty-three selected evaluated the development of new-onset atrial fibrillation. Ten studies [6, 8, 10, 11, 15, 26–28, 30, 35] found TAVI to be associated with a lower incidence, whereas one study [23] found TAVI to be less favourable than SAVR. Another study [31] found similar rates between TAVI and SAVR.

Five studies out of the thirty-three selected evaluated the development of atrial fibrillation. All studies [16, 24, 25, 29, 31] found TAVI patients to have lower rates of atrial fibrillation.

**Table 1:** Main effects of TAVI vs. SAVR for specific outcome measures

AUTOR, YEAR	SURGICAL RISK	EARLY MORTALITY	LATE MORTALITY	STROKE	CARDIAC MORTALITY	MYOCARDIAL INFARCTION	ALL-CAUSE MORTALITY	TIA
Wu YC, 2013	High	=	=	=		=		
Panchal, 2013	All	=	=	-	=	=		=
Nagaraja V, 2014	All	=	=	=		=		
Khan AR, 2016	High	=	=	-				
Villablanca PA, 2016	All		=	+				
Carnero-Alcázar M, 2017	Medium	=	=	=				
Wang Y, 2018	Low to intermediate		=	=		=		
Cheng X, 2019	All	+ (CKD)		+ (CKD)				
Ueshima D, 2019	Medium	+ (women)	+ (women)					
Ueshima D, 2019	Low	=	=			+		
Al-Abdouh A, 2020	Low			=			=	
Hofer F, 2020	Low	+	+					
Goel S, 2020	Low		=	=	=		=	
Kundu A, 2020	Low	=	=	=		=		
Vipparthy SC, 2020	Low	+	=	=		=		
Lou Y, 2020	Low	=	+	=		=		
Zhang D, 2020	Low to intermediate			=	=	+	=	+
Ueyama H, 2020	All	=	=					
D’Ascenzo F, 2021	All				=			
Matsuda Y, 2021	All			+				
Chen CG, 2022	Low				+ (1 y) = (2y)		+ (1 y) = (2y)	
Sá Pompeu M, 2022	All	+	-	=		=		
Ion AC, 2022	All			=	-			
Sakurai Y, 2022	All	=	-	=		=	-	
Barili F, 2022	All	+	-	+				
Ahmad Y, 2023	All	+(low risk) = (high risk)	=	+(low risk) = (high risk)				
Lerman, 2023	All			*=*	*-*	*=*	*=*	
Yokoyama, 2023	All			=	= (0–1 y) - (1–2 y) = (2–5 y)		+ (0–1 y) = (1–2 y) - (2–5 y)	
Jacquemyn, 2023	All	+	-					
Tariq, 2023	Low			+		+	+ (1y) = (2y)	
Heuts, 2023	All						-	
Sá Pompeu, 2023	All						-	
Llerena-Velastegui, 2024	Intermediate			=				

“+” TAVI has a significant better effect than SAVR technique, “=” there is no significant difference between TAVI and SAVR, “-“there is a significant worse effect than SAVR.

**Table 2:** Main effects of TAVI vs. SAVR for specific complications

AUTOR, YEAR	MAJOR BLEEDING	AKI	VASCULAR COMPLICATION	PACEMAKER IMPLANTATION	RESIDUAL AORTIC REGURGITATION	NEW-ONSET ATRIAL FIBRILLATION	MODERATE-SEVERE PARAVALVULAR LEAK	ATRIAL FIBRILLATION
Wu YC, 2013	= (REOPERATION)			-	-	+		
Panchal, 2013	+							
Nagaraja V, 2014		=		-	-	+	-	
Khan AR, 2016	+		-	-	-			
Villablanca PA, 2016	+	+	-	-	-			+
Carnero-Alcázar M, 2017	+	+	-	-	-			
Wang Y, 2018	+	+		-		+		
Cheng X, 2019		+ (CKD)	= (CKD)	- (CKD)				
Ueshima D, 2019							- (men)	
Ueshima D, 2019		+	-	-			-	
Al-Abdouh A, 2020	+	+		-		+	-	
Hofer F, 2020	+	+		-		+		
Goel S, 2020				=			-	+
Kundu A, 2020	+	+		-		+		
Vipparthy SC, 2020		+	-	-		+		
Lou Y, 2020	+	+	-	-	-		-	+
Zhang D, 2020		+	=	-	-	+		
Ueyama H, 2020				-				
D’Ascenzo F, 2021	+	+				+		
Matsuda Y, 2021								
Chen CG, 2022	+	+		-		+		
Sá Pompeu M, 2022		+	-	-				
Ion AC, 2022								
Sakurai Y, 2022	=	+	-	-				
Barili F, 2022								
Ahmad Y, 2023	-	-	+	+		-		
Lerman, 2023	+	+	-	-				+
Yokoyama, 2023				+ (0–1 years) + (1–2 years) = (2–5 years)				
Tariq, 2023						=		+

“+” TAVI has a significant better effect than SAVR technique, “=” there is no significant difference between TAVI and SAVR, “-“there is a significant worse effect than SAVR

## Conclusions

To the author’s knowledge, the present study is the first effort to summarize the evidence in an overview of SRs (OoSRs) focusing exclusively on secondary studies that have reported at least one review of clinical outcomes/process indicators. Hence this study summarizes the findings of existent literature reviews on the performance of TAVI in the last 10 years providing a rapid picture of the evolution of TAVI over time on the main KPIs. Our work shows that older reviews from 2013 to 2020 focused on high-risk patients, while since 2020 both high and low-risk patients were considered. There is a need to point out that reviews considering all risk classes of patients, did not report the results grouped by risk class. Accordingly, only one study reported the results for men and women separately. Regarding the included patients’ age, no reviews reported results broken down by age group. The authors recognize that by applying exclusion criteria, such as excluding systematic literature reviews containing observational studies or those solely focused on devices or imaging, there is a potential for narrowing the scope of the review. This narrowing could result in overlooking valuable insights from specific study designs or aspects of TAVI, which should be acknowledged as a limitation of the study. Still, the focus of this OoSRs was to identify and select relevant KPIs on selected outcomes and complications and not to compare devices.

AKI and new-onset atrial fibrillation showed a better performance overall for TAVI in comparison to SAVR. Other indicators suggest that TAVI presents a higher incidence of complications such as pacemaker implantation and vascular complications with respect to SAVR. Both early and late mortality showed no significant difference between the two procedures initially. For late mortality, a noticeable shift in the studies included emerges from 2022 onwards. Initially, both groups showed comparable mortality rates. However, in recent studies, TAVI demonstrates a higher mortality rate. Regarding patient risks, no distinctions were found among high-risk patients for both early and late mortality, while two studies observed lower early mortality rates for low-risk patients. The shift in included studies from 2022 onwards underscores the need for further investigation, particularly given the relative youth of the TAVI procedure. Medical devices and surgical procedures are historically associated with learning curves, meaning that the performance changes over time while the operators gain experience and refine their performing techniques. Additionally, advancements in imaging technologies, such as 3D echocardiography and intravascular ultrasound, have enhanced procedural guidance and accuracy.

The evolution of TAVI techniques over time has allowed for an expansion of the criteria for patient selection. Initially, only patients deemed inoperable for traditional open-heart surgery due to prohibitive surgical risks, comorbidity, and age underwent TAVI procedures. There has recently been an expansion of indications to intermediate and low-risk patients as well, based on clinical evidence and individual patient characteristics. Over time, a reduction in procedural complications and device-related complications such as paravalvular leakage and vascular complications, have been minimized with improved device designs and operator experience. Whereas surgical valves have a long-established track record, TAVI valves have been continuously improving in terms of design, durability, and longevity. Current research is focused on evaluating the durability and longevity of such valves in order to ensure maximum comfort to patients and lower rates of complications.

A long-term assessment of valve durability has only been possible in recent years, and few papers have investigated the differences between transcatheter and surgical bioprostheses. One study [40]revealed that TAVI valves exhibited a higher susceptibility to structural valve deterioration, while another study [41] indicated that the rate of moderate/severe structural valve deterioration was greater in SAVR cases, with similar rates of bioprosthetic valve failure between the two groups. It’s essential to acknowledge that TAVI is a newer technology compared to SAVR, thus still undergoing an evolution in terms of designs and enhancements.

Compared to open-heart surgery, TAVI requires smaller incisions and reduces the need for sternotomy. The minimally invasive nature of TAVI contributes to sustainability by minimizing patient discomfort and allowing for a quicker return to daily activities. Additional studies regarding the sustainability of the intervention and long-term durability of the implanted valves should be carried out in order to fully assess the potential for TAVI interventions. In conclusion, the evolution of TAVI seems an ongoing process, with current research and development, focused on improving patient outcomes, expanding indications, and addressing potential limitations.

### Electronic supplementary material

Below is the link to the electronic supplementary material.

Supplementary material 1

## Data Availability

All articles analyzed in this literature review are listed in the Reference section, and can be accessed coherently with the access policy of the publisher.
