# Supplementary material for: The evolution of TAVI performance overtime: an overview of systematic reviews
Source: BMC Cardiovasc Disord. 2024 Jun 21;24:314. doi: 10.1186/s12872-024-03980-2 (PMC11191264; doi:10.1186/s12872-024-03980-2)
Supplement: Supplementary file 1 — Supplementary material 1 [file 12872_2024_3980_MOESM1_ESM.docx]

**Appendix 1: Search strategy in Pubmed**

| **Search Number** | **Query** | **Search Details** | **Results** |
| --- | --- | --- | --- |
| 1 | TAVI[Title/Abstract] | "TAVI"[Title/Abstract] | 6,743 |
| 2 | Transcatheter aortic valve implantation[Title/Abstract] | "transcatheter aortic valve implantation"[Title/Abstract] | 8,089 |
| 3 | TAVR[Title/Abstract] | "TAVR"[Title/Abstract] | 6,786 |
| 4 | Transcatheter aortic valve replacement[Title/Abstract] | "transcatheter aortic valve replacement"[Title/Abstract] | 8,529 |
| 5 | Transcatheter aortic valve implantation[MeSH Terms] | "transcatheter aortic valve replacement"[MeSH Terms] | 11,880 |
| 6 | Review[Title/Abstract] | "Review"[Title/Abstract] | 2,305,230 |
| 7 | Meta analysis[Title/Abstract] | "meta analysis"[Title/Abstract] | 263,640 |
| 8 | Review[MeSH Terms] - Schema: all | Review[MeSH Terms] | 0 |
| 9 | Meta analysis[MeSH Terms] | "meta analysis as topic"[MeSH Terms] | 29,681 |
| 10 | #1 OR #2 OR #3 OR #4 OR #5 | "TAVI"[Title/Abstract] OR "transcatheter aortic valve implantation"[Title/Abstract] OR "TAVR"[Title/Abstract] OR "transcatheter aortic valve replacement"[Title/Abstract] OR "transcatheter aortic valve replacement"[MeSH Terms] | 19,063 |
| 11 | #6 OR #7 OR #8 OR #9 | "Review"[Title/Abstract] OR "meta analysis"[Title/Abstract] OR "meta analysis as topic"[MeSH Terms] | 2,414,046 |
| 12 | #10 AND #11 | ("TAVI"[Title/Abstract] OR "transcatheter aortic valve implantation"[Title/Abstract] OR "TAVR"[Title/Abstract] OR "transcatheter aortic valve replacement"[Title/Abstract] OR "transcatheter aortic valve replacement"[MeSH Terms]) AND ("Review"[Title/Abstract] OR "meta analysis"[Title/Abstract] OR "meta analysis as topic"[MeSH Terms]) | **2,448** |

**Appendix 2: Articles included**.

| **Author, Year** | **Time horizon** | **Study type** | **N° of patients** | **Age** | **Patient surgical risk** | **AMSTAR** | **Data type** |
| --- | --- | --- | --- | --- | --- | --- | --- |
| Carnero-Alcázar M, 2013 | <1 y | meta-analysis | 1,480 | Elderly (>65) | high | Low quality | Randomized clinical trials (RCTs) |
| Al-Abdouh A, 2013 | Up to 900 d | meta-analysis | 4,659 | all | all | High quality | Observational prospective/retrospective studies/pragmatic clinical trials |
| Khan AR, 2014 | Not reported | meta-analysis and systematic review | 13,130 | Elderly (>65) | all | High quality | Observational prospective/retrospective studies/pragmatic clinical trials |
| Hofer F, 2016 | <1 y | meta-analysis | 2,173 | Elderly (>65) | high | High quality | Randomized clinical trials (RCTs) |
| Cheng X, 2016 | Between 1 y and 5 y | literature search | 44,247 | all | all | High quality | Randomized clinical trials (RCTs) |
| Ueshima D, 2017 | Not reported | meta-analysis | 20,224 | all | medium | High quality | Randomized clinical trials (RCTs) |
| Villablanca PA, 2018 | > 2 y | meta-analysis | 4,355 | Elderly (>65) | low to intermediate | High quality | Randomized clinical trials (RCTs) |
| Goel S, 2019 | Not reported | meta-analysis | 9,619 | Elderly (>65) | all | Moderate quality | Observational prospective/ retrospective studies/pragmatic clinical trials |
| Kundu A, 2019 | Not reported | systematic review | NA | Elderly (>65) | medium | High quality | Observational prospective/retrospective studies/pragmatic clinical trials |
| Ueshima D, 2019 | <1 y | meta-analysis | 9,805 | Elderly (>65) | low | High quality | Observational prospective/retrospective studies/pragmatic clinical trials |
| Vipparthy SC, 2020 | Not reported | meta-analysis | 2,698 | Elderly (>65) | low | Low quality | Randomized clinical trials (RCTs) |
| Wu YC, 2020 | Between 1 y and 5 y | meta-analysis and systematic review | 2,887 | Elderly (>65) | low | Moderate quality | Randomized clinical trials (RCTs) |
| Lou Y, 2020 | Between 1 y and 5 y | meta-analysis | 6,293 | Elderly (>65) | low | Low quality | Randomized clinical trials (RCTs) |
| Zhang D, 2020 | Between 1 y and 5 y | meta-analysis | 2,953 | Elderly (>65) | low | High quality | Randomized clinical trials (RCTs) |
| Panchal H, 2020 | Between 3 m and 5 y | meta-analysis | 27,956 | Elderly (>65) | low | High quality | Randomized clinical trials (RCTs) |
| Wang Y, 2020 | < 2 y | meta-analysis | 12,467 | Elderly (>65) | low | High quality | Randomized clinical trials (RCTs) |
| Nagaraja V, 2020 | Beetwen 30 d and 2 y | meta-analysis and systematic review | 6,929 | Elderly (>65) | low to intermediate | Moderate quality | Randomized clinical trials (RCTs) |
| D'Ascenzo F, 2020 | Between 1 and 2 y | meta-analysis | 10,300 | Elderly (>65) | all | Low quality | Randomized clinical trials (RCTs) |
| Ueyama H, 2021 | Between 1 y and 5 y | meta-analysis | 10,359 | Elderly (>65) | all | Moderate quality | Randomized clinical trials (RCTs) |
| Matsuda Y, 2021 | Not reported | meta-analysis and systematic review | 26,270 | all | all | Low quality | Observational prospective/retrospective studies/pragmatic clinical trials |
| Chen CG, 2022 | 2 y | meta-analysis | 2,644 | all | low | Moderate quality | Randomized clinical trials (RCTs) |
| Sá M, 2022 | Up to 4 y | meta-analysis | 33,286 | Elderly (>65) | all | Low quality | Observational prospective/retrospective studies/pragmatic clinical trials |
| Ion AC, 2022 | Not reported | meta-analysis | 14,601 | all | all | Low quality | Observational prospective/retrospective studies/pragmatic clinical trials |
| Sakurai Y, 2022 | Up to 3 y | meta-analysis and systematic review | 104,22 | Elderly (>65) | all | Moderate quality | Observational prospective/retrospective studies/pragmatic clinical trials |
| Barili F, 2022 | Between 1 y and 5 y | meta-analysis | 7,770 | all | all | High quality | Randomized clinical trials (RCTs) |
| Ahmad Y, 2023 | Between 1 y an 8 y | meta-analysis | 8,698 | all | all | Low quality | Randomized clinical trials (RCTs) |
| Lerman T, 2023 | Between 1 y and 5 y | meta-analysis and systematic review | 8,780 | Elderly (>65) | all | High quality | Randomized clinical trials (RCTs) |
| Yokoyama Y, 2023 | Between 1 y and 5 y | meta-analysis | 8,885 | all | all | Moderate quality | Randomized clinical trials (RCTs) |
| Jacquemyn X, 2023 | Between 1 y and 5 y | meta-analysis | 15,212 | all | all | Low quality | Observational prospective/retrospective studies/pragmatic clinical trials |
| Tariq M, 2023 | Between 1 y and 8 y | meta-analysis | 3,865 | Elderly (>65) | low | Moderate quality | Randomized clinical trials (RCTs) |
| Heuts S, 2023 | Between 1 y and 5 y | meta-analysis and systematic review | 8,698 | Elderly (>65) | all | Moderate quality | Randomized clinical trials (RCTs) |
| Sá Pompeu M, 2023 | Between 1 y and 8 y | meta-analysis | 5,444 | Elderly (>65) | low | Moderate quality | Observational prospective/retrospective studies/pragmatic clinical trials |
| Llerena-Velastegui J, 2024 | Between 1 y and 5 y | meta-analysis and systematic review | 10,658 | Elderly (>65) | intermediate | Moderate quality | Randomized clinical trials (RCTs) |
